# Radiomics using computed tomography to predict CD73 expression and prognosis of colorectal cancer liver metastases

**DOI:** 10.1186/s12967-023-04175-7

**Published:** 2023-07-27

**Authors:** Ralph Saber, David Henault, Nouredin Messaoudi, Rolando Rebolledo, Emmanuel Montagnon, Geneviève Soucy, John Stagg, An Tang, Simon Turcotte, Samuel Kadoury

**Affiliations:** 1 MedICAL Laboratory, Polytechnique Montréal, Montréal, H3T 1J4 Canada; 2 Imaging and Engineering Axis, Centre de recherche du Centre Hospitalier de l’Université de Montréal/Institut du cancer de Montréal, 900 rue Saint-Denis R10.430, Montréal, QC H2X 0A9 Canada; 3 Cancer Axis, Centre de recherche du Centre Hospitalier de l’Université de Montréal/Institut du cancer de Montréal, 900 rue Saint-Denis, Room R10.430, Montréal, QC H2X 0A9 Canada; 4 Hepato-Pancreato-Biliary Surgery and Liver Transplantation Service, Centre hospitalier de l’Université de Montréal, 1000, rue Saint-Denis, Montréal, QC H2X 0C1 Canada; 5 Department of Surgery, Vrije Universiteit Brussel (VUB), Universitair Ziekenhuis Brussel (UZ Brussel) and Europe Hospitals, Brussels, Belgium; 6 Pahology Department, Centre hospitalier de l’Université de Montréal, 1000, rue Saint-Denis, Montréal, QC H2X 0C1 Canada; 7 Department of Computer and Software Engineering, Institute of Biomedical Engineering, Polytechnique Montréal, Montréal, H3T 1J4 Canada; 8 Department of Radiology, Radiation Oncology and Nuclear Medicine, Université de Montréal, Montréal, H3T 1J4 Canada

**Keywords:** Cancer, Immune checkpoint, Adenosine pathway, CD73, Radiomic biomarker, Interpretable machine learning

## Abstract

**Background:**

Finding a noninvasive radiomic surrogate of tumor immune features could help identify patients more likely to respond to novel immune checkpoint inhibitors. Particularly, CD73 is an ectonucleotidase that catalyzes the breakdown of extracellular AMP into immunosuppressive adenosine, which can be blocked by therapeutic antibodies. High CD73 expression in colorectal cancer liver metastasis (CRLM) resected with curative intent is associated with early recurrence and shorter patient survival. The aim of this study was hence to evaluate whether machine learning analysis of preoperative liver CT-scan could estimate high vs low CD73 expression in CRLM and whether such radiomic score would have a prognostic significance.

**Methods:**

We trained an Attentive Interpretable Tabular Learning (TabNet) model to predict, from preoperative CT images, stratified expression levels of CD73 (CD73^High^ vs. CD73^Low^) assessed by immunofluorescence (IF) on tissue microarrays. Radiomic features were extracted from 160 segmented CRLM of 122 patients with matched IF data, preprocessed and used to train the predictive model. We applied a five-fold cross-validation and validated the performance on a hold-out test set.

**Results:**

TabNet provided areas under the receiver operating characteristic curve of 0.95 (95% CI 0.87 to 1.0) and 0.79 (0.65 to 0.92) on the training and hold-out test sets respectively, and outperformed other machine learning models. The TabNet-derived score, termed rad-CD73, was positively correlated with CD73 histological expression in matched CRLM (Spearman’s *ρ* = 0.6004; *P* < 0.0001). The median time to recurrence (TTR) and disease-specific survival (DSS) after CRLM resection in rad-CD73^High^ vs rad-CD73^Low^ patients was 13.0 vs 23.6 months (*P* = 0.0098) and 53.4 vs 126.0 months (*P* = 0.0222), respectively. The prognostic value of rad-CD73 was independent of the standard clinical risk score, for both TTR (HR = 2.11, 95% CI 1.30 to 3.45, *P* < 0.005) and DSS (HR = 1.88, 95% CI 1.11 to 3.18, *P* = 0.020).

**Conclusions:**

Our findings reveal promising results for non-invasive CT-scan-based prediction of CD73 expression in CRLM and warrant further validation as to whether rad-CD73 could assist oncologists as a biomarker of prognosis and response to immunotherapies targeting the adenosine pathway.

**Supplementary Information:**

The online version contains supplementary material available at 10.1186/s12967-023-04175-7.

## Background

Liver metastases is the most common site of colorectal cancer progression, a malignancy that remains within the top leading causes of cancer-related deaths [1]. Complete resection of colorectal liver metastases (CRLM) combined with systemic chemotherapy, has a curative potential, but approximately 80% of patients recur [2]. Currently, no biomarkers can help identify patients at high risk of early recurrence after CRLM resection, for whom surgery may be futile, who may benefit from adjuvant chemotherapy [3], and who should be followed up more closely based on the risk of recurrence. Consequently, a significant number of patients are burdened by potential complications, side-effects and toxicities associated with treatments not informed by expected prognosis, without overall survival benefits.

Accumulating data support that the immune features of CRLM may be more significantly associated with recurrence and survival after resection, independently from clinical, pathological and tumor genomic features [4]. Extracellular adenosine in the tumor microenvironment (TME) appears as an immunosuppressive mechanism of particular relevance to CRLM microenvironment. The Ecto-5′-nucleotidase, or CD73, expressed by cancer and stromal cells within CRLM, is a rate-limiting enzyme that enhances the breakdown of ATP-derived extracellular adenosine monophosphate into immunosupressive adenosine [5]. The latter binds to the A2A and A2B receptors expressed by T cells, Natural Killer cells, and other immune cells and inhibits their anti-tumoral cytotoxic functions [6]. High intratumoral CD73 expression is strongly associated with poor prognosis in both primary CRC [7] and CRLM [8].

The current histologic assessment of CD73 expression in CRLM, like other histologically-derived biomarkers, is however obtained only after resection of CRLM. Histological-based markers are also not ideal to asses pre-operatively via biopsy given their intratumoral heterogeneous expression [9]. The field still lacks noninvasive CRLM biomarkers of immune features to guide prognostication, which may also, at term, help identify patients most likely to benefit from immunotherapy. Features of computed tomography (CT) images are widely available and part of routine clinical practice, both at baseline and on follow-up examinations for assessment of treatment response, and radiomics biomarkers obtained from these images could help clinical decisions early in the treatment course. Current developments in artificial intelligence span almost all conventional medical image analysis tasks, including the detection and segmentation of anatomical structures, classification and registration of medical images [10]. Radiomics, consisting in extracting quantifiable features from medical images, could detect not only macroscopic characteristics, but also hidden genomic and proteomic properties involved in biological processes [11, 12]. Radiomics studies have shown promising results in the diagnosis of liver diseases, including benign diseases and primary and secondary malignancies, cancer staging and grading and the prediction of patient clinical outcomes such as response to therapy [13, 14]. Concerning the liver, contrast-enhanced CT-scan features have been shown able to detect non-alcoholic steatohepatitis [15], to identify the histological growth pattern of CRLM [16], and to predict response to FOLFOX-based chemotherapy in untreated CRLM patients [17]. To our knowledge, however, CT-scan image features have not been associated with immune features in CRLM [18].

The goal of this study was to test whether we could train a deep learning model with radiomic features extracted from preoperative CT images, to predict postoperative CD73 expression in resected CRLM, and to test whether a probabilistic score predicted by the deep learning model, termed rad-CD73, was associated with patient oncological prognosis.

## Methods

### Study population

This study received the approval of the Institutional Review Board (No. 19.185 for patient consent for biobanking and database; No. 18.023 for project specific with radiomics). We retrospectively studied a cohort of 215 patients who underwent complete resection of CRLM at the Centre Hospitalier de l’Université de Montréal between 2011 and 2014 and were prospectively followed beyond recurrence and until death for clinicopathological and imaging data in a registry. Clinical annotations included demographics, dates and types of all treatments received, and the Clinical Risk Score, calculated by the addition of one point for each of the following features: disease-free interval between the diagnosis of primary tumor and liver metastases of < 12 months; number of metastases > 1; pre-operative carcinoembryonic antigen (CEA) level > 200 ng/mL; largest metastasis > 5 cm; and lymph node positive primary tumor [19]. A liver pathologist reviewed hematoxylin and eosin whole slides of all cases to assess resection margins, the presence of tertiary lymphoid structures, the degree of necrosis and pathological response to preoperative chemotherapy [20].

The inclusion criteria were as follows: (1) CRLM confirmed by histopathological analysis; (2) complete resection of CRLM with curative intent; (3) preoperative, intravenous contrast-enhanced abdominal CT scan available; and (4) CD73 immunofluorescent quantification staining performed. Patients were excluded from the analysis if: (1) preoperative CT images were insufficient to perform feature analysis; (2) CRLM was not visible on the preoperative CT scan or was calcified; (3) histopathological results could not be associated with CT image of a given CRLM on the basis of its description in pathology report and review of the CT-scan. After applying the exclusion criteria, 160 CRLM resected in 122 patients were available for further analysis.

### Evaluation of CD73 expression

CD73 expression in CRLM quantified by immunofluorescence were previously generated in this cohort [8]. Briefly, we built tissue microarrays (TMA) using six 0.6 µm TMA cores per CRLM, with up to three CRLM per patient, using FFPE blocks after hematoxylin and eosin review of viable tumor areas by a pathologist and trained resident. We optimized a multiplex immunofluorescence panel to concurrently detect CD73, cytokeratins to compartmentalize stromal and cancer cell expression patterns, and DAPI for nuclear staining of viable cells. Standard deparaffinization and rehydratation protocols were used, followed by antigen retrieval (Dako S1699) in sub-boiling conditions for 40 min, and protein-block (Dako X0909), specific staining with primary antibodies against CD73 (Abcam ab91086, 1:300 dilution) and cytokeratins 8/18 (Dako IR094, 1:2 dilution). We used an anti-mouse IgG1 Alexa-Fluor 647 (Life technology, A21240; 1/800) and anti-rabbit Alexa-Fluor 488 (Life technology, A21206; 1/400) as secondary antibodies, DAPI, and mounted the slides with ProLong Gold (ThermoFisher). Slides were digitalized at 20 × with NanoZoomer-XR (Hamamatsu) and core images imported with TMA maps and identifiers into Visiomorph v.6 software (Visiopharm) for automated quantification. For each core, the percent surface area containing CD73^+^ cells (expression above background) over the surface area containing all viable cells was calculated, as well as the mean fluorescence intensity in each core. For each CRLM, a mean value was calculated from its corresponding six cores. Patients were stratified into two classes (CD73^High^ and CD73^Low^) using the median CD73^+^ positive area score across all CRLM evaluated in this cohort as a cutoff value (3.8%). In patients with more than one CRLM, the mean CD73 was used to classify patients as low or high.

### Image preprocessing and radiomics workflow

We analyzed the last contrast-enhanced CT images obtained prior to surgery for CRLM resection and acquired in portal venous phase. The images had a cross-sectional volume size of 512 $\times$ 512, a mean in-plane resolution of 0.72 mm^2^ (range = [0.56, 0.98] mm^2^) and a mean in-depth resolution of 2.26 mm (range = [0.80, 5.0] mm). Images were resampled to isotropic resolution (1 × 1 × 1 mm^3^) so as to obtain a uniform pixel spacing within the dataset. An automated segmentation algorithm [21] was used to segment CRLM lesions. The segmentation model consists of two convolutional neural networks trained end-to-end in order to jointly segment the liver and the lesions within. A manual examination and refinement of the ensuing 3D segmentations was subsequently carried out by liver radiologists to verify the quality of the volumes of interest to be included in the analysis.

In a subsequent step, radiomic features were extracted from the volumes of interest using PyRadiomics v3.0.1 toolbox [22]. From each image/tumor mask pair, we extracted 107 radiomics features consisting of 18 first-order statistics, 14 shape features and 75 textural features. First-order statistics are histogram-derived features characterizing the distribution of voxel values within the tumor. Shape features encode the 3D shape and size of the region of interest. These features are calculated from approximated shapes, inferred using triangle meshes generated from binary masks using the marching cube technique. Details on the marching cubes algorithm used to build meshes are presented in the Pyradiomics documentation [22]. Thirdly, textural features are derived from predefined matrices and aim to construe the spatial arrangement of voxel intensity values within the lesion. The textural matrices included are the gray-level co-occurrence matrix (GLCM), the gray level dependence matrix (GLDM), the gray-level run length matrix (GLRLM), the gray-level size zone matrix (GLSZM) and the neighboring gray tone difference matrix (NGTDM).

The resulting feature set was standardized for the sake of obtaining a null mean and a unit standard deviation across instances. The least absolute shrinkage and selection operator (LASSO) [23] was used to select the most salient features. The rationale behind this step is to apply an initial coarse dimensionality reduction in order to discard irrelevant features that would otherwise introduce noise into the training process and mislead the model. The LASSO λ hyperparameter (λ = 0.249) was determined by applying five-fold cross validation using the mean squared error as an objective function. Each lesion segmented on CT was matched to the corresponding CRLM in the constructed TMA, using their pathology report block number.

### TabNet training and evaluation

In this study, we trained an Attentive Interpretable Tabular Learning (TabNet) model [24] to predict the stratified CD73 expression levels (CD73^High^ vs. CD73^Low^). TabNet is a deep learning model that incorporates multiple stages of attention modules within its architecture (Additional file 1: Fig. S1). The attention mechanism is based on two transformer blocks: an attentive transformer and a feature transformer. The attentive transformer allows to compute learnable masks, which are used to select the most salient set of features at each step. It also incorporates a prior scale which encodes the degree to which features have been used in the previous steps. The feature transformer processes the filtered features through shared and decision step-specific layers. The outputs of the different decision steps are linearly combined to form the model’s encoder output. Finally, a fully connected layer processes the encoder’s output to obtain the overall output of the model. In order to compare TabNet’s performance with other machine learning models, we also trained an XGBoost, a random forest (RF), a support vector machine (SVM) with a linear kernel function, and a logistic regression (LR) model to perform the same task.

For TabNet and the other baseline models, we first divided the dataset into a training set (125 lesions) and a hold-out test set (35 lesions) for independent validation using data from our center. We then performed a five-fold cross-validation on the first subset. The dataset was hence divided into five separate folds on five iterations and in each iteration, four folds were used for training and the fifth was used for validation (never seen at training). Once cross-validation was completed, an external validation was performed on the hold-out test set. All splits were applied randomly and on a patient-level. In other words, lesions belonging to the same patient were assigned to the same subset in order to ensure that no overlap or information leakage occur between the training and the testing sets. TabNet was trained for 100 epochs on an NVIDIA GeForce GTX TITAN Xp 12 GB with a batch size of 64 and a binary cross-entropy loss function. Adam optimizer [25] was used with a learning rate of 0.02 decaying by 10% after 50 epochs. The area under the receiver operating characteristic curve (AUC) was used to compare the performance of the models.

### Model interpretability analysis

Two types of interpretability analyses were carried out in this study: global and local interpretability. In the former case, we attempted to holistically describe the model’s behavior by running a subsequent analysis on the model’s predictions. To this end, we adopted the Shapley Additive Explanations (SHAP) technique [26]. SHAP is an additive feature attribution algorithm that intends to compute, for every feature, a Shapley value which mirrors the contribution of that feature to the model’s final predictions. In this work, we utilized the Kernel SHAP method, which approximates the Shapley values as being the coefficients of a weighted linear regression model, built from a set of sample coalitions. After computing an average Shapley value for each feature over all instances, the features were ranked according to their average Shapley values. Additionally, we made use of the inherent interpretability of the TabNet architecture to provide instance-level explanations of the model’s predictions. To do so, visualizations of the selected feature masks were generated and analyzed, in order to identify the most salient ones for each instance used by the model. The visualization of TabNet’s selected feature importance served two main purposes: (1) to acquire a local explanation of the model’s predictions for each instance and (2) to examine whether the predictive behavior projected by the model’s architecture, was concordant with SHAP results.

### Statistical analysis

We used the Wilcoxon rank sum test and Fisher’s exact test for numerical and categorical variables, respectively. Spearman’s correlation coefficient was applied to evaluate correlations. The decision curve analysis [27] was performed using the Python dcurves v0.0.3 package by plotting the net benefit of the predicted biomarker for different threshold probability values [range = (0–1)], given as the minimum probability for which additional testing is recommended. The line representing the “all lesions are CD73^Low^” hypothesis and the curve representing the “all lesions are CD73^High^” hypotheses were also plotted for comparison purposes. Survival curves were generated with the Kaplan–Meier method and compared with the log-rank test. Disease-specific survival, for which deaths due to causes other than cancer progression were censored observations, and time to recurrence, were computed from the date of the first hepatectomy. Patients with missing data on the first surgery were excluded from the survival analysis. For survival analysis purposes, TabNet class probabilities, termed rad-CD73, were derived and stratified into rad-CD73^High^ and rad-CD73^Low^ by setting a cut-off value equal to the lower tertile (rad-CD73 > 0.362) based on the distribution of the rad-CD73 score (Additional file 1: Fig. S2), which was consistent with the optimal p-value cut-off proposed by the X-tile software (0.383). For patients with multiple lesions, predicted rad-CD73 scores were averaged across all lesions. Univariate and multivariate Cox proportional hazards regression models were used to generate hazards ratios (HR) with 95% confidence intervals. A two-sided p-value < 0.05 was considered statistically significant. Statistical analyses were conducted using Python Scipy v1.5.3, Python Lifelines v0.27.1 and R Survival v3.4 packages.

## Results

**Fig. 1:**
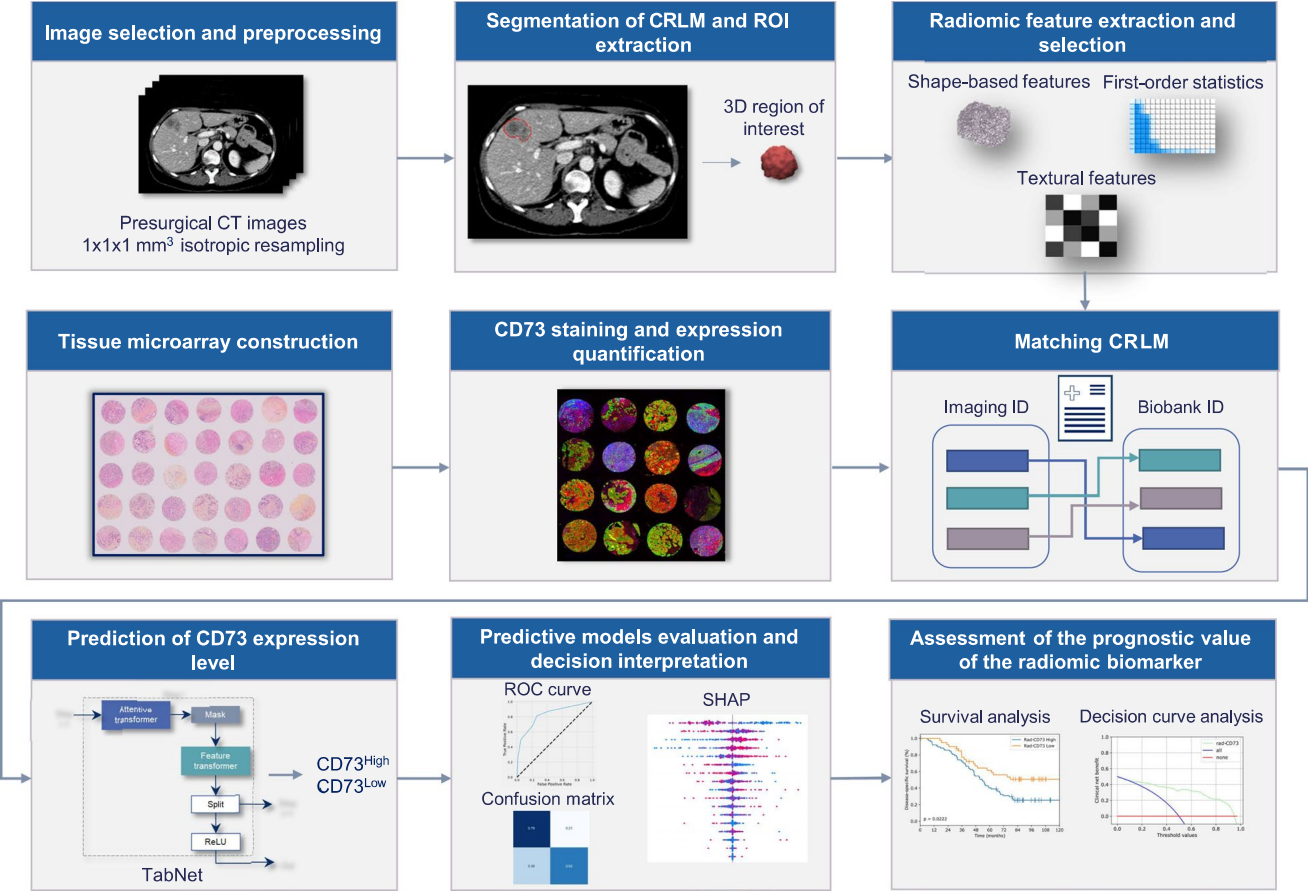
General workflow. Isotropic spatial resampling was applied on preoperative CT-scan images to segment 160 colorectal liver metastases (CRLM) resected in 122 colorectal cancer (CRC) patients who underwent partial hepatectomy. Matching CRLM, identified by their pathology report block numbers and anatomical description, were included in tissue microarrays for automated quantification of CD73 intra-tumoral expression by immunofluorescence. Radiomic features were extracted from the resulting three-dimensional regions of interest (ROI) (18 first-order statistics, 14 shape features and 75 textural features) and preprocessed. Subsequently, an Attentive Interpretable Tabular Learning (TabNet) model was trained to predict CD73 expression dichotomized as CD73^High^ vs. CD73^Low^ based on the surface area expressing CD73 over the total surface of assessable tissue. The model was then evaluated, and its predictions were interpreted using ROC curve and the Shapley Additive Explanations technique (SHAP). The association between radiomic CD73 (rad-CD73) and oncological survival outcomes was analyzed by Kaplan–Meier and logrank test

The general workflow of the planned analysis is depicted in Fig. 1.

### Patient cohort

The clinicopathological characteristics of the 122 patients treated for resectable CRLM are summarized in Table 1. Mean patient age was 63.4 years (35 to 84), and male patients predominated (63.9%). Preoperative chemotherapy was administered in 78.7% of the patients, consisting of four to six cycles of folfox-based regimen for the vast majority of patients (not shown)**.** Most patients (81.1%) also received chemotherapy after resection of CRLM. Approximately half of the patients were treated for multiple metastasis with a mean number of two (range 1 to 10) and a mean diameter of 4.1 cm (range 1.0 to 20 cm). Based on the composite Clinical Risk Score [19], 38.5% of patients were at high risk of recurrence and death from cancer progression. At time of analysis, the median follow-up was of 57.0 months, during which time 76.2% of patients had recurred and 64.8% died of disease progression.

**Table 1:** Patient clinicopathological characteristics and correlation with rad-CD73

Variable	All dataset (N = 122)	rad-CD73^High^ (n = 79)	rad-CD73^Low^ (n = 43)	p*-value*
Age at hepatectomy (Mean, range)	(63.4, 35–84)	(63.6, 35–84)	(63.2, 44–81)	0.894
≤ 65 years	66 (54.1)	42 (53.2)	24 (55.8)	
> 65 years	56 (45.9)	37 (46.8)	19 (44.2)	
Gender				0.430
Male	78 (63.9)	48 (60.8)	30 (69.8)	
Female	44 (36.1)	31 (39.2)	13 (30.2)	
Number of metastases (Mean, range)	(2.0, 1–10)	(2.1, 1–10)	(1.9, 1–5)	0.643
Single	59 (48.4)	41 (51.9)	18 (41.9)	
Multiple	63 (51.6)	38 (48.1)	25 (58.1)	
Diameter of largest metastasis (Mean, range)	(4.1, 1.0–20.0)	(4.3, 1.0–20.0)	(3.8, 1.3–11.0)	0.830
≤ 5 cm	93 (76.2)	59 (74.7)	34 (79.1)	
> 5 cm	29 (23.8)	20 (25.3)	9 (20.9)	
KRAS status				0.735
Wild-type	27 (61.4)	18 (58.1)	9 (69.2)	
Mutated	17 (38.6)	13 (41.9)	4 (30.8)	
CEA level				0.287
≤ 200 ng/mL	118 (97.5)	77 (98.7)	41 (95.3)	
> 200 ng/mL	3 (2.5)	1 (1.3)	2 (4.7)	
Clinical risk score^a^				0.017
Low risk (0–2)	72 (61.5)	53 (69.7)	19 (46.3)	
High risk (3–5)	45 (38.5)	23 (30.3)	22 (53.7)	
Margin liver resection				0.325
Negative	111 (91.0)	70 (88.6)	41 (95.3)	
Positive	11 (9.0)	9 (11.4)	2 (4.7)	
Tertiary lymphoid structure				0.775
No	107 (87.7)	70 (88.6)	37 (86.0)	
Yes	15 (12.3)	9 (11.4)	6 (14.0)	
Necrosis (Mean, range)	(12.3, 0.0–81.5)	(13.2, 0–81.5)	(10.7, 0.0–61.8)	0.358
≤ 25%	104 (85.2)	67 (84.8)	37 (86.0)	
> 25%	18 (14.8)	12 (15.2)	6 (14.0)	
Primary tumor				0.140
Left sided	82 (69.5)	58 (74.4)	24 (60.0)	
Right sided	36 (30.5)	20 (25.6)	16 (40.0)	
pT category				0.190
pT1-pT3	93 (83.0)	56 (78.9)	37 (90.2)	
pT4	19 (17.0)	15 (21.1)	4 (9.8)	
pN category				0.116
pN0	43 (35.8)	32 (41.0)	11 (26.2)	
pN +	77 (64.2)	46 (59.0)	31 (73.8)	
Preop chemotherapy				1.000
No	26 (21.3)	17 (21.5)	9 (20.9)	
Yes	96 (78.7)	62 (78.5)	34 (79.1)	
Tumor regression grade^b^				0.575
1–2	16 (13.1)	9 (11.4)	7 (16.3)	
3–4–5	106 (86.9)	70 (88.6)	36 (83.7)	
Extrahepatic recurrence				0.806
No	32 (36.0)	24 (37.5)	8 (32.0)	
Yes	57 (64.0)	40 (62.5)	17 (68.0)	
Disease-free interval between primary and liver met				
< 12 months	88 (72.1)	52 (65.8)	36 (83.7)	0.037
≥ 12 months	34 (27.9)	27 (34.2)	7 (16.3)	

*CEA* carcinoembryonic antigen, *CD* cluster of differentiation, *T* tumor, *N* node

^a^Clinical Risk Score calculated by the addition of one point for the following features: disease-free interval between the diagnosis of primary tumor and liver metastases < 12 months; number of metastases > 1; pre-operative CEA level > 200 ng/mL; largest metastasis > 5 cm; and lymph node positive primary tumor[19]

^b^Measure of histopathological response to neoadjuvant chemotherapy as defined by Rubbia-Brandt et al.[20]

### Prediction of CD73 expression from preoperative CT images

**Fig. 2:**
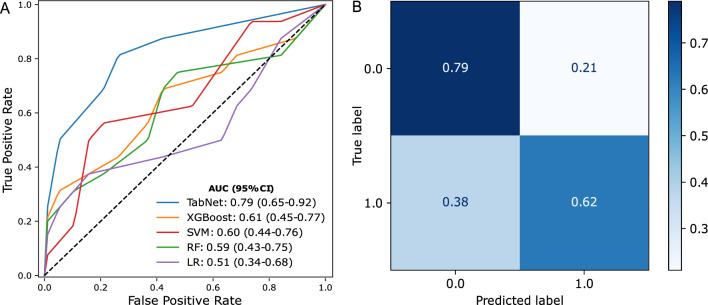
Performance of TabNet on the hold-out test set.** A** Comparison of the ROC curves of TabNet with the other baseline models. TabNet outperformed all the trained baseline models as mirrored by the area under the ROC curves (AUC). **B** Confusion matrix of TabNet for CD73 classification. LR, Logistic Regression; RF, Random Forest; SVM, Support Vector Machine; TabNet, Attentive Interpretable Tabular Learning; XGBoost, Extreme Gradient Boosting

In the training cohort, the ability of the TabNet model to classify CD73^High^ vs. CD73^Low^ lesions was shown to have an AUC of 0.95 (95% confidence interval: 0.87- 1.0). The accuracy, sensitivity and specificity were 0.85, 0.91 and 0.79, respectively. Table 2 summarizes the performance of different models on the hold-out test set. Moreover, TabNet exhibited a high predictive performance on the hold-out test set with an AUC of 0.79 (0.65–0.92). The test set accuracy, sensitivity and specificity were 0.71, 0.63 and 0.79, respectively. Figure 2 depicts the receiver operating characteristic curve and the confusion matrix of the model on the hold-out test set. The model exhibited balanced sensitivity and specificity values, and no class bias was observed. This is reflected by the distribution of the true positives and true negatives in the matrix. We also compared TabNet generalization capability with other machine learning models, which outperformed XGBoost, RF, SVM and LR models. Even though the XGBoost model achieved a satisfactory AUC of 0.61 (0.45–0.77), TabNet outclassed it with a high margin.

**Table 2:** Performance of the different models on the hold-out test set

Model	AUC	Accuracy	Sensitivity	Specificity	F1 score
TabNet	0.79 (95% CI 0.65–0.92)	0.71	0.63	0.79	0.67
XGBoost	0.61 (95% CI 0.45–0.77)	0.60	0.69	0.53	0.61
SVM	0.60 (95% CI 0.44–0.76)	0.49	0.94	0.11	0.63
RF	0.59 (95% CI 0.43–0.75)	0.63	0.75	0.53	0.65
LR	0.51 (95% CI 0.34–0.68)	0.46	0.63	0.32	0.51

*AUC* area under the curve, *TabNet* attentive interpretable tabular learning, *XGBoost* extreme gradient boosting; *SVM* support vector machine, *RF* random forest, *LR* logistic regression

**Fig. 3:**
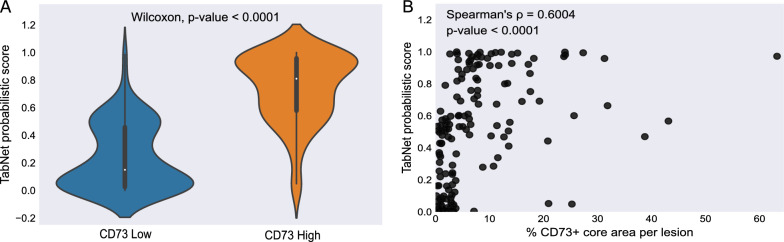
Assessment of the predicted TabNet probabilistic score, rad-CD73. **A** Violin plot depicting the distribution of rad-CD73 score by CD73 expression level. A statistically significant difference was observed in the radiomic score between the CD73^High^ and CD73^Low^ groups (Wilcoxon signed rank test; *P* < 0.0001). **B** Spearman’s correlation between rad-CD73 and the actual CD73 expression (the percentage of CD73 positive area per metastasis)

**Fig. 4:**
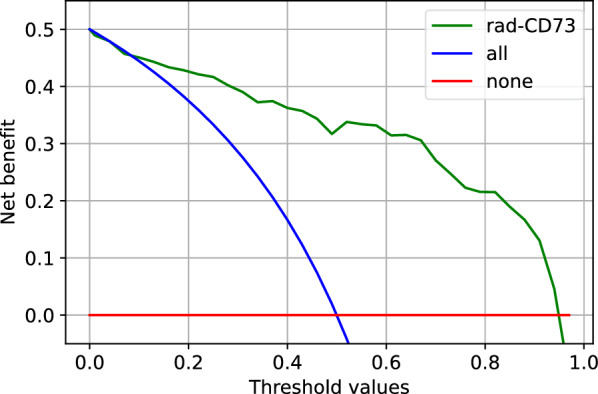
Decision curve analysis. The red line shows the treat none as CD73^High^ approach while the blue line shows the treat all as CD73^High^ approach. For threshold values greater than 0.08, rad-CD73 had a higher net benefit than both the “treat all” and “treat none” approaches

A significant difference was observed in the predicted TabNet rad-CD73 scores between CD73^High^ and CD73^Low^ lesions (Wilcoxon signed rank test, *P* < 0.0001) (Fig. 3A). Rad-CD73 scores were positively correlated with CD73 histological expression measured by IF (Spearman’s *ρ* = 0.600, *P* < 0.0001) (Fig. 3B). With the goal of assessing the clinical utility of the predicted rad-CD73 score, we performed a decision curve analysis and compared the net benefit of using rad-CD73 with the “treat all as CD73^High^” and the “treat none as CD73^High^” strategies. For probability thresholds higher than 0.08, rad-CD73 had a higher net benefit than both the “treat all” and “treat none” approaches (Fig. 4).

### Interpretability of the predictive model

**Fig. 5:**
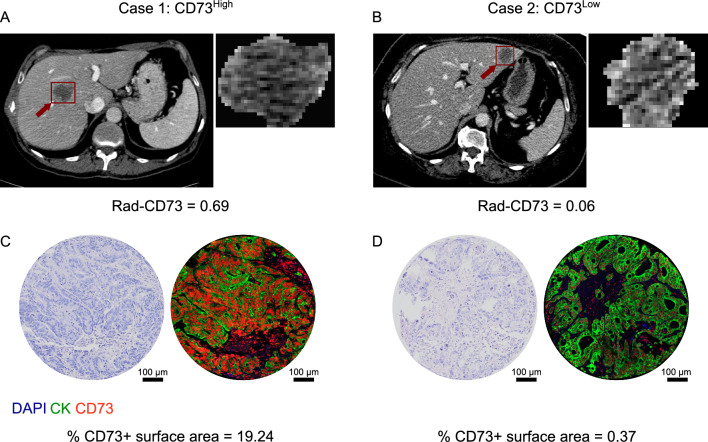
Representative cases with high and low CD73 expression.** A** A preoperative CT image of a CD73^High^ CRLM (arrow) in the right hemiliver. **B** A preoperative CT image of a CD73^Low^ CRLM (arrow) in the left hemiliver. **C**, **D** Representative tissue microarray cores of the high **C** and low **D** CD73 expression of the respective CRLM. Hematoxylin and eosin staining (left) shows the integrity and general architecture of the tissue. Immunofluorescent staining (right) shows cell nucleus (blue, DAPI), CD73 expression (red), and the cancer cells with the pan-cytokeratins epithelial cell marker (green). Scale bars represent 100 µm

**Fig. 6:**
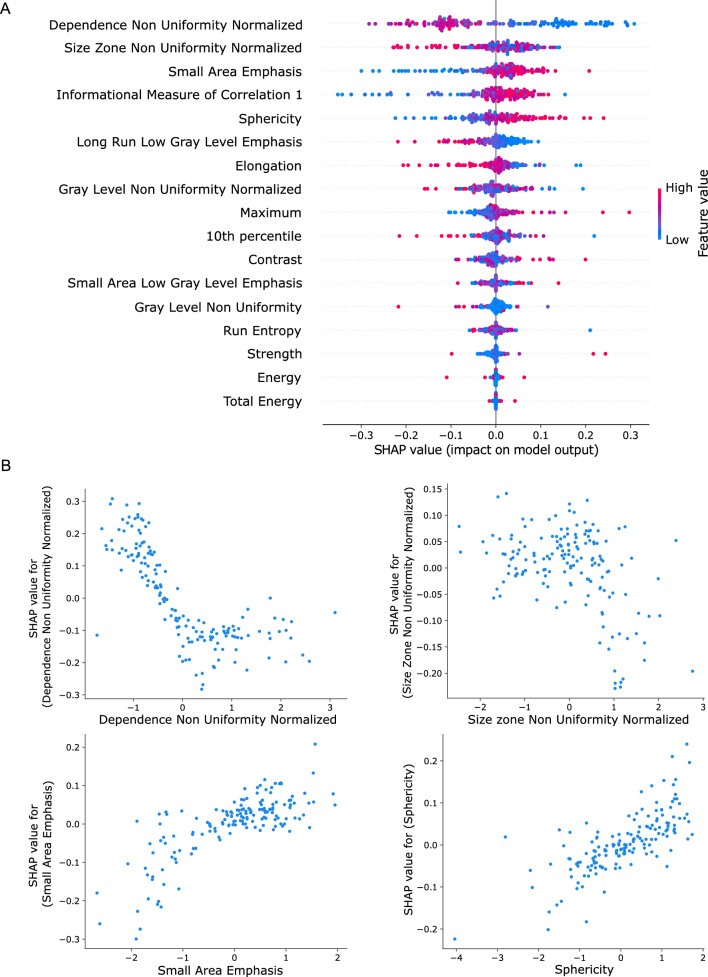
TabNet global interpretability analysis using the Shapley Additive Explanations (SHAP) technique. **A** Summary plot listing radiomics features from top to bottom in a decreasing order of their impact on the model’s decision. The top four most significant features were texture-related features. **B** Variation of the SHAP value of four selected features with respect to the actual feature value. Homogeneous textures were associated with a higher CD73 expression, as mirrored by the effect of the top-ranking features

Figure 5 shows representative CT-scan images and corresponding histological images and CD73 IF expression of two CRLM cases with high and low CD73 expression, respectively. Case 1 (left) represents a CRLM with a high CD73 expression (high red IF signal, % CD73 + surface area = 19.24). Concordantly, the corresponding rad-CD73 probabilistic score was 0.69. On the other hand, a low rad-CD73 score (0.06) was attributed to the CRLM of case 2 (right) having a low CD73 IF expression (% CD73 + surface area = 0.37). This finding supported that different CT-scan features could be observed between CD73^High^ and CD73^Low^ CRLM, with homogeneity in CT tumor segmentation in case 1 and heterogeneity in the CT tumor segmentation in case 2. To improve the interpretation of TabNet’s predictions, we applied the SHAP technique and studied the features that contributed the most to the model’s outputs. Figure 6 shows that distinct features had different impact on the model’s output, mirrored by its average Shapley value. Amongst the top five features selected by SHAP, four were textural, reflecting the importance of texture-related characteristics of the lesions in predicting CD73 expression level.

The feature with the highest Shapley value was the “Dependence Non Uniformity Normalized” (DNUN), a textural feature computed using the GLDM matrix. The DNUN encodes the heterogeneity in terms of the dependence throughout the lesion: a high DNUN reveals that the image contains regions with disparate dependence levels. The dependence is a term reflecting whether the gray level of a given voxel is dependent on those of the neighboring ones. A region with a low dependence is hence formed of voxels with comparable gray levels whereas the voxels of a high dependence region exhibit discrepancies in their gray levels. SHAP results show that a low DNUN value had a positive impact on the model’s output, prompting an increase in the predicted rad-CD73 score.

Similarly, the textural feature “Size Zone Non Uniformity Normalized” (SZNUN), which encodes the heterogeneity in the size zone volumes of a lesion defined as areas with a constant gray level, had an effect comparable the DNUN on TabNet predictions. The “Small Area Emphasis” (SAE) and the “Informational Measure of Correlation 1” (IMC1) came in third and fourth, respectively. The SAE reflects the prevalence of small zones while the IMC1 encodes the complexity of the textures. Both features had a positive impact on the model’s output; a higher feature value was corresponded to a higher Shapley value. These findings show that the model had the tendency to output high rad-CD73 scores for lesions exhibiting finer textures.

**Fig. 7:**
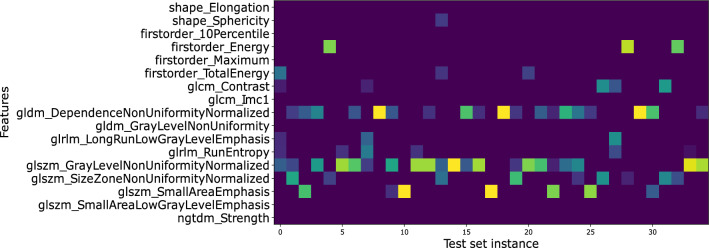
Visualization of TabNet learnable mask from the third decision step. The dependence non uniformity normalized (DNUN) feature, designated as the top 1 feature in SHAP analysis, was selected by the learnable mask among the most salient features for almost all test set instances. Brighter colors indicate higher feature importance

Interestingly, the shape of the lesions also had an impact, albeit less prominent, on the model’s behavior. In fact, SHAP results reveal that spherical lesions were associated with high CD73 expression levels. Finally, Fig. 7 shows that the DNUN was selected for almost all test set instances among the most salient features in TabNet’s third feature selection stage. This finding was consistent with SHAP, as the DNUN had the highest average Shapley value.

### Association of rad-CD73 with clinicopathological characteristics and oncological outcomes

We then analyzed the potential clinical significance of rad-CD73 high vs low status. As shown in Table 1, the proportions of rad-CD73^High^ and rad-CD73^Low^ patients were generally similar according to most clinicopathological characteristics, including CRLM size and number, having received chemotherapy or not prior to liver resection, and pathological response to pre-operative chemotherapy. There was hence no statistically significant difference in CRLM diameter (*P* = 0.830), whether the primary tumor is located in the right or left colon (*P* = 0.140), the KRAS mutation status (*P* = 0.735) and the CEA level (*P* = 0.287) between rad-CD73^High^ and rad-CD73^Low^ CRLM. More patients with rad-CD73^High^ CRLM were however found in those who were diagnosed with liver metastases less than 12 months after the diagnosis of primary CRC (*P* = 0.037). Although this criterion is one of those constituting the composite clinical risk score (CRS), there were less rad-CD73^High^ patients in those classified as higher risk of recurrence (CRS score 3, 4 or 5).

**Fig. 8:**
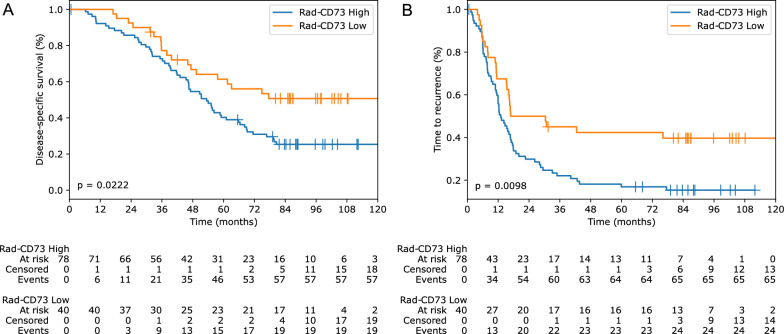
Prognostic value of rad-CD73 in colorectal liver metastases (CRLM). **A** Disease-specific survival according to rad-CD73 and **B** time-to-recurrence after the initial complete surgical resection of CRLM. In patients with more than one CRLM, the mean rad-CD73 was used to classify patients as low or high. The lower tertile was used as a cut-off value (rad-CD73 > 0.362)

By univariate analysis, rad-CD73 high vs low status was significantly associated with TTR and DSS, as well as expected clinicopathological features such as the primary tumor depth of invasion (pT stage), high pre-operative CEA, and the composite CRS (Table 3). In the initial immunohistochemical study of CD73 expression in this patient cohort [8], patients with high intratumoral CD73 expression had significantly shorter median TTR compared to low CD73 (11.0 vs 46.4 months) and DSS (19.0 vs. 61.5 months), independent of conventional clinicopathological variables by multivariate analyses. Consistent with the worse prognosis observed in patients bearing tumor with high intratumoral CD73 expression assessed by immunohistochemistry [8, 28–31], patients with rad-CD73^High^ CRLM had a shorter median TTR of 13.0 months compared to 23.6 months in rad-CD73^Low^ CRLM patients (*P* = 0.0098). Consistently, the median DSS of rad-CD73^High^ CRLM patients was 53.4 months compared to 126.0 months in rad-CD73^Low^ CRLM patients (*P* = 0.0222) (Fig. 8). Consistent with the lack of positive association observed between the CRS and rad-CD73, multivariate modeling supported that the prognostic value of rad-CD73 was independent of the CRS for both TTR and DSS (Table 4).

**Table 3:** Univariate analyses of outcomes according to clinicopathological variables and rad-CD73

	Disease-specific survival		Time-to-recurrence	
	HR (95% CI)	p-value	HR (95% CI)	p-value
Age at hepatectomy (≤ 65 vs > 65 years)	0.88 (0.56–1.39)	0.58	0.70 (0.46–1.07)	0.10
Gender (male vs female)	1.16 (0.72–1.84)	0.54	0.97 (0.63–1.51)	0.91
Number of metastases (single vs multiple)	1.37 (0.87–2.16)	0.17	1.50 (0.98–2.29)	0.06
Diameter of largest metastasis (≤ 5 vs > 5 cm)	1.43 (0.87–2.37)	0.16	1.44 (0.90–2.30)	0.13
Disease-free interval < 12 months (no vs yes)	1.52 (0.90–2.58)	0.12	1.43 (0.89–2.28)	0.14
CEA level (≤ 200 vs > 200 ng/mL)	4.38 (1.34–14.38)	0.01	5.12 (1.57–16.62)	0.01
Tertiary lymphoid structure (absent vs present)	0.86 (0.43–1.73)	0.68	0.95 (0.49–1.84)	0.88
Liver resection margin (negative vs positive for cancer cells)	1.06 (0.50–2.22)	0.89	1.10 (0.55–2.18)	0.79
Necrosis (≤ 25 vs > 25%)	1.18 (0.63–2.18)	0.61	1.02 (0.57–1.80)	0.96
Primary tumor (left vs right)	0.79 (0.48–1.28)	0.33	0.87 (0.55–1.37)	0.54
pT category (T1-T2-T3 vs T4)	2.05 (1.16–3.59)	0.01	2.51 (1.45–4.34)	< 0.005
pN category (N0 vs N +)	1.25 (0.77–2.02)	0.37	1.30 (0.84–2.01)	0.25
Pre-operative chemotherapy (no vs yes)	1.73 (0.91–3.27)	0.09	1.73 (0.99–3.03)	0.05
Tumor regression grade (1–2 vs 3–4–5)	0.84 (0.44–1.61)	0.61	1.31 (0.68–2.52)	0.43
KRAS status (wild-type vs mutated)	1.18 (0.61–2.28)	0.63	2.23 (1.14–4.37)	0.02
Clinical Risk Score (0–1–2 vs 3–4–5)	1.60 (1.01–2.51)	0.04	1.97 (1.29–3.03)	< 0.005
rad-CD73 (low vs high)	1.80 (1.08–3.00)	0.02	1.84 (1.15–2.95)	0.01

*HR* hazard ratio, *CI* confidence interval

**Table 4:** Multivariate analyses of outcomes according to clinical risk score and rad-CD73

	Disease-specific survival		Time-to-recurrence	
	HR (95% CI)	p-value	HR (95% CI)	p-value
Clinical risk score (0–1–2 vs 3–4–5)	1.83 (1.15–2.92)	0.01	2.40 (1.54–3.74)	< 0.005
rad-CD73 (low vs high)	1.88 (1.11–3.18)	0.02	2.11 (1.30–3.45)	< 0.005

*HR* hazard ratio, *CI* confidence interval

## Discussion

In this study, we developed a noninvasive imaging surrogate of CD73 by leveraging state-of-the-art deep learning techniques trained with radiomic features. Despite recent progress in prognostication based on immune features of CRLM resected with curative intent [4], there are no noninvasive immune biomarkers that ultimately may guide clinical decision making. To our knowledge, this is the first work developing and testing a machine learning tool to predict immunosuppressive CD73 expression from diagnostic CT images. The proposed model exhibited good performance in classifying CRLM lesions into CD73^High^ and CD73^Low^ groups. We also demonstrated that the predicted rad-CD73 score was highly correlated with the actual expression as measured in vitro by immunohistochemistry. Moreover, the clinical significance of rad-CD73 was supported by its association with patient prognosis.

Radiomics has achieved major breakthroughs in recent years in precision oncology and has paved the way for individualized patient care. Its clinical applications include disease diagnosis, prognosis and treatment planning [32, 33]. It could hence be applied for cancer detection, allowing for a noninvasive differentiation between benign and malignant neoplasms and consequently minimizing the unwarranted collection of tissue samples. Moreover, radiomics was coupled with conventional diagnostic tools in order to augment their sensitivity to detect diseases early in their development [34]. Finally, it was shown to be able to forecast oncological outcomes of patients such as survival, recurrence, response to adjuvant therapy and metastatic progression [35]. The main advantage of radiomics over conventional techniques is that it provides a holistic and noninvasive assessment of the tissues, as opposed to more invasive histopathological tissue analysis methods and RNA sequencing which require biopsies taken from tumor regions. Its performance is hence less affected by the tumoral heterogeneity associated with the biopsy site. Moreover, it is less labor intensive and allows a quicker profiling of the patients than existing diagnostic tools. Nevertheless, the translation of radiomics to the clinic has been hindered by some challenges including the current retrospective design of the majority of the radiomics studies and the “black box” nature of the predictive models. Therefore, radiomics should be considered as a promising complementary tool for personalized treatment to be validated prospectively.

In the past few years, several efforts have been made for the interpretation of the results produced by machine learning models, which are still considered as black boxes. Model interpretability is particularly important in the medical oncology field to ensure traceability and informed clinical decision-making. Interpretability techniques could be divided into two major categories: model-specific and model-agnostic [36]. The former interpretability is acquired in models that are conceived to be inherently explainable through attention mechanisms for instance, which provides feedback on regions of focus in deep learning [37, 38]. On the other hand, model-agnostic interpretability techniques are usually implemented separately and do not depend on the model’s architecture. While the latter involves the implementation of an additional step, it presents the advantage of being applicable to a variety of models. In this work, we tested both techniques. We first trained a TabNet model and sought to understand its instance-level decisions by visualizing its feature selection masks. In a post-hoc analysis, we applied SHAP technique to decipher its overall behavior. The study revealed that the most salient features for the prediction of CD73 expression were texture-related. Moreover, textural heterogeneity was associated with a lower CD73 expression and inversely applicable. This was mirrored by the impact of the DNUN, the SZNUN and the IMC1 on the model’s predictions. These findings are in line with several previous studies associating textural features with response to immunotherapy [39]. Tang et al. [40] found a cluster of non-small cell lung cancer patients exhibiting concurrent tumoral heterogeneity and high CD3 T cell infiltration. Yoon et al. [41] showed that type 2 helper T cells were associated with high variance and IMC, mirroring lesion heterogeneity. On the other hand, Sun et al. [42] found, in a study combining several cancer sites, that a high CD8 score, indicative of inflammatory infiltrate, was associated with homogeneous lesion appearances. They also attributed heterogeneity in pixel intensities to convoluted underlying processes such as excessive disorderly tumor vascularization. CT-scan image features may also be enhanced by the use of nanoparticles, characterized by a high permeability and retention in tumors. Several nanoparticles have been tested [43], including silver nanoparticles [44]. In this context, Devkota et al. [45] demonstrated that radiomics features, extracted from nanoparticle contrast-enhanced CT rather than conventional imaging, were better suited for the prediction of response to cellular immunotherapy.

Immunotherapy has become a mainstay in the treatment of several advanced malignancies. This has motivated several researchers to leverage radiomics to forecast response to immunotherapeutic agents [46–48] either by predicting established biomarkers within the tumor microenvironment or by attempting to directly associate imaging features with patient outcomes, such as radiological response, survival and time-to-recurrence. However, the vast majority of the conducted studies focus on non-small cell lung cancer given the universal availability of chest images and the proven effectiveness of immune checkpoint inhibitors (ICI) in advanced lung cancer. The application of radiomics in CRLM immuno-oncology remains vastly unexplored. While prior work on radiomics of CRLM have focused on response to chemotherapy as measured by the tumor regression grade [49] or the RECIST criteria [50, 51], and tumor histological features such as the histological growth patterns [16], we aimed to develop and validate a radiomic immune marker for CRLM.

While first generation ICI such as anti-programmed cell death protein 1 (PD1), anti-PD1 ligand and anti-cytotoxic T lymphocyte antigen 4 have proven to be effective in some cancers [52], 95% of metastatic CRC are refractory to these immunotherapies [53, 54]. The underlying mechanisms driving these poor outcomes include tumor heterogeneity and the coexistence of complex immune escape mechanisms within the hepatic tumor microenvironment. Because of the immunosuppressive nature of the adenosine pathway, adenosinergic molecules are now being explored for the development of novel therapeutic agents [55]. In particular, CD73 ectonucleotidase plays a major role in the generation of immunosuppressive adenosine and has recently emerged as a novel immunotherapeutic target that can be blocked by monoclonal antibodies, while adenosine receptor inhibitors are also being tested in early phase trials [56, 57].

Overall, the clinical use of high rad-CD73 on CT-scan imaging of patients with resectable CRLM, as it may identify a subset of patients with earlier recurrence, death, and higher intratumoral CD73 expression, could be tested prospectively in many ways to determine whether: a) closer follow-up after CRLM resection could lead to earlier treatment of recurrence and survival benefits; b) adjuvant systemic therapy after CRLM resection could reduce the risk of recurrence and death; and c) it can predict the efficacy of patients more likely to respond to anti-CD73 or adenosine receptor inhibitors.

Our work has some limitations notwithstanding. First, because this is a single-center investigation, it will be important to verify the reproducibility of the results by testing the model on a cohort of patients recruited in different institutions. Leveraging epidemiologically diversified databases is of equal importance in order to minimize any bias that could be introduced by unrepresentative datasets. Second, the sample size is relatively limited to deploy deep learning models. Nevertheless, radiomic pipelines have the advantage of being more transparent than end-to-end black box models due to the interpretability of radiomic features. Third, our work does not take into account the dynamic aspect of the tumor microenvironment, with varying delays between the preoperative CT and the histopathological analysis from one patient to another. Future studies should take into account the temporal fluctuations when training artificial intelligence tools to characterize tumor biology. This is markedly true in radiomics-based analyses since medical images could be leveraged in longitudinal studies as a result of their omnipresence.

## Conclusions

In this study, we introduced a deep learning pipeline for the prediction of CD73 expression in curatively resected CRLM from preoperative CT images. The conceived rad-CD73 biomarker could serve as a noninvasive, fast and low cost tool to identify candidates for targeted immunotherapy. Due to its association with patient prognosis, it could also be leveraged to assist oncologists to personalize the need for adjuvant treatments and the intensity of follow-up strategies. The generalizability of the model needs to be validated on independent, large and epidemiologically diverse cohorts, and its impact on clinical decision making will need to be tested prospectively.

## Supplementary Information

**Additional file 1: Fig. S1**. TabNet model architecture depicts the architecture of the deep learning model used in this work. **Fig. S2.** Distribution of the predicted TabNet probabilistic score, rad-CD73, across the patients.

## Data Availability

All raw tissue microarray images and associated marker quantification and patient clinicopathological characteristics are available upon request from the study authors.

## Abbreviations

AUC: Area under the receiver operating characteristic curve
CEA: Carcinoembryonic antigen
CRLM: Colorectal cancer liver metastases
CRC: Colorectal cancer
CRS: Clinical risk score
CT: Computed tomography
DNUN: Dependence non uniformity normalized
GLCM: Gray-level co-occurrence matrix
GLDM: Gray level dependence matrix
GLRLM: Gray-level run length matrix
GLSZM: Gray-level size zone matrix
HR: Hazards ratios
ICI: Immune checkpoint inhibitors
IMC: Informational measure of correlation
LASSO: Least absolute shrinkage and selection operator
LR: Logistic regression
NGTDM: Neighboring gray tone difference matrix
PD1: Programmed cell death protein 1
RF: Random forest
SVM: Support vector machine
SAE: Small area emphasis
SHAP: Shapley additive explanations
SZNUN: Size zone non uniformity normalized
TabNet: Attentive interpretable tabular learning
TMA: Tissue microarrays
