# Supplementary material for: Radiomics using computed tomography to predict CD73 expression and prognosis of colorectal cancer liver metastases
Source: J Transl Med. 2023 Jul 27;21:507. doi: 10.1186/s12967-023-04175-7 (PMC10375693; doi:10.1186/s12967-023-04175-7)
Supplement: Supplementary file 1 — Additional file 1: Fig. S1. TabNet model architecture depicts the architecture of the deep learning model used in this work. Fig. S2. Distribution of the predicted TabNet probabilistic score, rad-CD73, across the patients. [file 12967_2023_4175_MOESM1_ESM.docx]

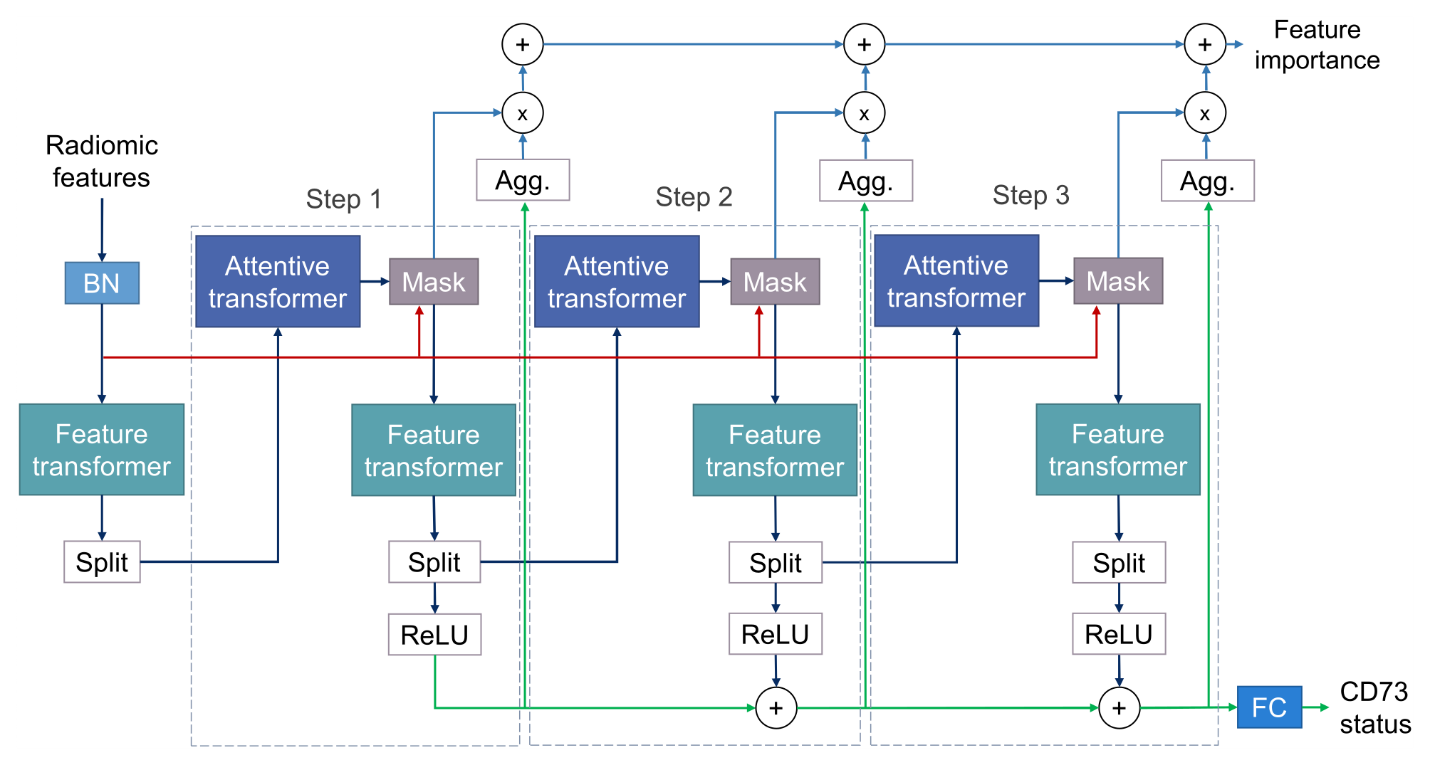


**Fig. S1 TabNet model architecture.** The model’s two main components are the attentive transformer and the feature transformer. The attentive transformer generates a learnable mask which allows to select an optimal set of features at a given decision step. The masking performed by the selection mask is specific to each step. The filtered features at a given step are sent to the feature transformer and the result is then used to process the following step and to generate the output of the current step. The outputs of the decision steps are linearly combined and a fully connected layer is finally added to obtain the model’s output. BN, Batch Normalization; ReLU, Rectified Linear Unit; Agg., Aggregation; FC, Fully Connected layer.


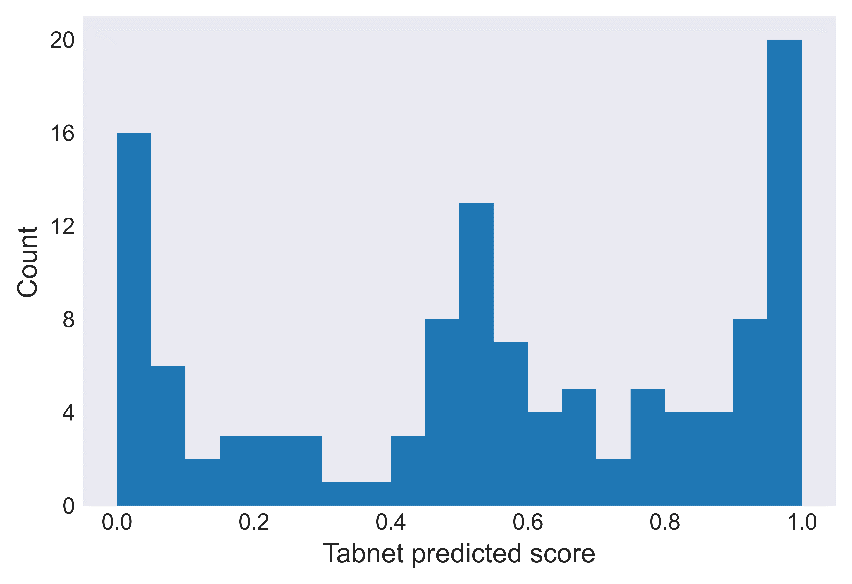


**Fig. S2 Distribution of the predicted TabNet probabilistic score, rad-CD73, across the patients.** A trimodal distribution was observed for rad-CD73. The X-tile software returned an outcome-based cutpoint of 0.383 for this distribution (close to the first tertile value of 0.362)
